# Blood urea nitrogen to serum albumin ratio: a novel mortality indicator in intensive care unit patients with coronary heart disease

**DOI:** 10.1038/s41598-024-58090-y

**Published:** 2024-03-29

**Authors:** Lingzhi Zhang, Muqi Xing, Qi Yu, Zihan Li, Yilin Tong, Wenyuan Li

**Affiliations:** Center of Clinical Big Data and Analytics of The Second Affiliated Hospital and Department of Big Data in Health Science School of Public Health, Zhejiang University School of Medicine, Hangzhou, 310058 Zhejiang China

**Keywords:** Biomarkers, Cardiology, Diseases, Health care, Medical research, Risk factors

## Abstract

The blood urea nitrogen to albumin ratio (BAR) has been demonstrated as a prognostic factor in sepsis and respiratory diseases, yet its role in severe coronary heart disease (CHD) remains unexplored. This retrospective study, utilizing data from the Medical Information Mart for Intensive Care-IV database, included 4254 CHD patients, predominantly male (63.54%), with a median age of 74 years (IQR 64–83). Primary outcomes included in-hospital, 28-day and 1-year all-cause mortality after ICU admission. The Kaplan–Meier curves, Cox regression analysis, multivariable restricted cubic spline regression were employed to assess association between BAR index and mortality. In-hospital, within 28-day and 1-year mortality rates were 16.93%, 20.76% and 38.11%, respectively. Multivariable Cox proportional hazards analysis revealed associations between the increased BAR index and higher in-hospital mortality (HR 1.11, 95% CI 1.02–1.21), 28-day mortality (HR 1.17, 95% CI 1.08–1.27) and 1-year mortality (HR 1.23, 95% CI 1.16–1.31). Non-linear relationships were observed for 28-day and 1-year mortality with increasing BAR index (both *P* for non-linearity < 0.05). Elevated BAR index was a predictor for mortality in ICU patients with CHD, offering potential value for early high-risk patient identification and proactive management by clinicians.

## Introduction

Coronary heart disease (CHD) remains a leading global cause of mortality, despite the advances in coronary revascularization and significant progress in secondary preventive treatments^1^. In the United States, CHD accounts for approximately one-third of all adult deaths^2^. CHD patients in the ICU grappling with present intricate clinical profiles stemming from diverse etiologies, accompanied by a heightened propensity for complications. Yet, the prognosis for CHD patients in the ICU has been a subject of limited studied^3^. The urgent need for readily applicable and straightforward prognostic indicators is paramount for CHD patients, particularly those in critical condition.

A pivotal metric, blood urea nitrogen (BUN), emerges as a metabolic byproduct of protein metabolism, offering insights into renal function^4^. Beyond serving as a gauge for glomerular filtration rate (GFR), BUN captures nuances such as renal hypoperfusion, diminished cardiac output, and neurohumoral activity^5–7^. An array of investigations has underscored the correlation between BUN levels and prognostic outcomes across diverse CVD contexts^8–10^. However, factors such as advanced age, gastrointestinal bleeding, corticosteroid interventions, and high-protein diets can confound BUN elevation by intensifying urea production^7,11^. Consequently, a solitary reliance on BUN measurements may fall short in ensuring robust predictive precision. Concurrently, the significance of serum albumin comes to the fore, attributed to its pivotal roles in upholding osmotic equilibrium, facilitating molecular conveyance, antioxidative actions, anti-inflammatory properties, and stabilization of endothelial function^12,13^. Notably, serum albumin levels can be influenced by patient nutritional status, hepatic synthesis, and catabolism dynamics^14,15^. Therefore, the exclusive utilization of albumin levels as prognostic indicators also confronts intrinsic limitations.

The construct of the blood urea nitrogen to albumin ratio (BAR) has garnered attention due to its potential as a predictive marker across various diseases, mainly including respiratory ailments^16^ and septic conditions^17^. However, a conspicuous gap exists in the current body of knowledge: the absence of investigations into the nexus between BAR and prognosis in CHD patients in the ICU.

Thus, this study aimed to investigate the association between BAR and all-cause mortality in CHD patients by using the fourth-generation Medical Information Mart for Intensive Care (MIMIC-IV, version 2.2) database.

## Results

**Figure 1:**
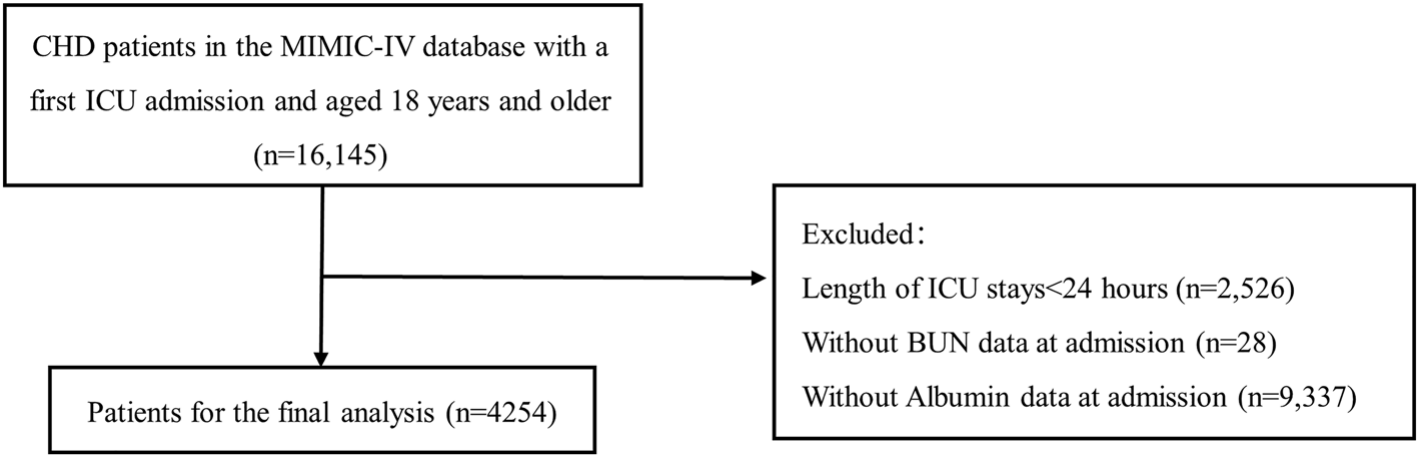
Flow chart of study population. *CHD* coronary heart disease, *ICU* intensive care unit, *BUN* blood urea nitrogen.

A total of 4254 patients with coronary heart disease in the ICU were ultimately enrolled in this study (Fig. 1). Among them, the median age of the patients was 74 (IQR 64–83) years, and 2703 (63.54%) were male. The median BAR index for all the patients was 8.00 (IQR 4.98–14.01). The in-hospital, 28-day and 1-year all-cause mortality rate were 16.93%, 20.76% and 38.11%, respectively (Table 1).

**Table 1:** Baseline characteristics of critical patients with CHD grouped according to quartiles of BAR index.

Characteristic	Overall	Q1	Q2	Q3	Q4
	(N = 4254)	(N = 1064)	(N = 1058)	(N = 1068)	(N = 1064)
Age (years)	74 (64, 83)	69 (59, 78)	75 (65, 83)	76 (67, 84)	75 (66, 84)
Gender					
Male, n (%)	2703 (63.54)	642 (60.34)	678 (64.08)	657 (61.52)	726 (68.23)
Female, n (%)	1551 (36.46)	422 (39.66)	380 (35.92)	411 (38.48)	338 (31.77)
Ethnicity					
White ethnicity, n (%)	2806 (65.96)	669 (62.88)	725 (68.53)	702 (65.73)	710 (66.73)
Black ethnicity, n (%)	330 (7.76)	88 (8.27)	61 (5.76)	87 (8.15)	94 (8.83)
Other, n (%)	1118 (26.28)	307 (28.85)	272 (25.71)	279 (26.12)	260 (24.44)
Severity score					
SOFA score	5 (3, 8)	3 (1, 5)	4 (2, 7)	6 (4, 9)	7 (5, 10)
SAPS II	39 (31, 49)	31 (25, 38)	36 (29, 44)	42 (36, 51)	48 (40, 58)
OASIS	34 (28, 40)	31 (25, 37)	33 (28, 39)	34 (29, 41)	36 (30, 43)
Comorbidities					
Hypertension, n (%)	1753 (41.21)	629 (59.12)	504 (47.64)	377 (35.30)	243 (22.84)
Diabetes, n (%)	1734 (40.76)	334 (31.39)	390 (36.86)	469 (43.91)	541 (50.85)
Obesity, n (%)	497 (11.68)	111 (10.43)	114 (10.78)	136 (12.73)	136 (12.78)
Heart failure, n (%)	2128 (50.02)	330 (31.02)	534 (50.47)	608 (56.93)	656 (61.65)
AKI, n (%)	1992 (46.83)	152 (14.29)	366 (34.59)	640 (59.93)	834 (78.38)
Sepsis3, n (%)	2658 (62.48)	506 (47.56)	618 (58.41)	729 (68.26)	805 (75.66)
Vital signs					
Heart rate (bpm)	81.81 (71.41, 93.11)	79.56 (70.28, 90.49)	80.62 (70.62, 91.45)	83.60 (72.69, 95.76)	83.98 (72.31, 95.21)
SBP (mmHg)	113.87 (104.95, 126.52)	117.78 (107.44, 131.06)	115.18 (105.12, 127.75)	112.98 (104.58, 123.87)	110.74 (102.93, 122.16)
DBP (mmHg)	60.91 (54.52, 68.13)	64.46 (58.08, 71.76)	61.58 (54.92, 68.60)	60.04 (53.91, 66.35)	58.00 (52.13, 64.73)
MBP (mmHg)	76.08 (70.12, 83.41)	80.10 (74.17, 88.01)	77.20 (71.23, 83.99)	74.66 (68.96, 81.43)	72.65 (67.31, 78.97)
Laboratory test					
Hemoglobin (g/dL)	10.73 (9.25, 12.30)	11.73 (10.30, 13.25)	11.20 (9.80, 12.70)	10.44 (9.03, 11.80)	9.60 (8.40, 10.90)
Platelet (K/uL)	192.00 (141.33, 251.50)	204.67 (158.68, 260.81)	188.58 (146.56, 247.50)	188.88 (132.94, 246.75)	180.50 (125.67, 254.87)
WBC (K/uL)	11.30 (8.41, 15.45)	10.76 (8.20, 13.61)	11.40 (8.30, 15.36)	11.35 (8.50, 15.97)	12.23 (8.65, 17.11)
ALT (IU/L)	28.00 (17.00, 61.50)	24.25 (16.80, 46.00)	28.00 (17.00, 60.50)	29.00 (17.00, 67.95)	33.00 (17.00, 80.13)
Creatinine (mg/dL)	1.22 (0.90, 1.98)	0.80 (0.70, 1.00)	1.08 (0.87, 1.33)	1.50 (1.10, 1.93)	2.58 (1.82, 3.59)
Glucose (mg/dL)	137.00 (112.00, 179.00)	128.17 (110.00, 155.71)	137.00 (113.00, 177.38)	141.00 (115.15, 185.00)	142.23 (111.33, 198.08)
Bicarbonate (mmol/L)	22.50 (19.50, 25.00)	23.67 (21.67, 25.67)	23.00 (20.53, 25.50)	22.00 (19.00, 24.69)	20.25 (17.25, 23.35)
Sodium (mEq/L)	138.50 (135.80, 141.00)	138.37 (136.33, 140.33)	138.67 (136.25, 141.00)	138.50 (135.67, 141.00)	138.33 (134.50, 141.25)
Potassium (mEq/L)	4.20 (3.90, 4.65)	4.05 (3.80, 4.33)	4.13 (3.85, 4.50)	4.30 (3.93, 4.71)	4.55 (4.05, 5.10)
Calcium (mg/dL)	8.38 (7.88, 8.85)	8.57 (8.10, 8.95)	8.45 (7.95, 8.90)	8.27 (7.80, 8.75)	8.23 (7.70, 8.71)
Albumin (g/dL)	3.35 (2.90, 3.80)	3.70 (3.30, 4.05)	3.50 (3.10, 3.90)	3.20 (2.80, 3.60)	2.91 (2.55, 3.40)
BUN (mg/dL)	26.20 (17.00, 43.00)	13.67 (11.00, 16.00)	21.69 (19.00, 24.66)	32.67 (27.67, 38.62)	61.23 (49.00, 80.54)
Events					
LOS hospital (days)	7.85 (4.91, 13.28)	6.77 (4.43, 10.88)	7.73 (4.86, 12.87)	8.21 (5.12, 14.01)	9.07 (5.38, 15.00)
LOS ICU (days)	2.87 (1.79, 5.08)	2.48 (1.67, 4.31)	2.82 (1.71, 4.81)	2.92 (1.82, 5.24)	3.16 (1.94, 5.97)
Hospital mortality, n (%)	720 (16.93)	76 (7.14)	126 (11.91)	218 (20.41)	300 (28.20)
28-days mortality, n (%)	883 (20.76)	95 (8.93)	162 (15.31)	259 (24.25)	367 (34.49)
1-year mortality, n (%)	1621 (38.11)	197 (18.52)	331 (31.29)	482 (45.13)	611 (57.42)

*CHD* coronary heart disease, *BAR* blood urea nitrogen-to-albumin ratio, *SOFA* sequential organ failure assessment, *SAPS II* simplifed acute physiological score II, *OASIS* Oxford acute severity of illness score, *AKI* acute kidney injury, *SBP* systolic blood pressure, *DBP* diastolic blood pressure, *MBP* mean blood pressure, *WBC* white blood cell, *ALT* alanine aminotransferase, *BUN* blood urea nitrogen, *LOS* length of stay, *ICU* intensive care unit.

### Baseline characteristics

Baseline** c**haracteristics both for the entire enrolled population and the stratified groups were presented in Table 1. Patients were divided into four groups according to the quartiles of the BAR index levels: Q1: 0.55–4.98; Q2: 4.98–8.00; Q3: 8.00–14.00; Q4: 14.00–52.90. Patients with higher BAR index generally had higher severity of illness scores on admission, higher prevalence of diabetes, heart failure, AKI and sepsis, higher heart rate and higher levels of WBC, ALT, creatinine, glucose, serum potassium compared to the lower group. We noticed that patients with higher BAR index had lower SBP, DBP, MBP and lower levels of bicarbonate and serum calcium. As the BAR index increased, there was a gradual rise in: ICU length of stay (2.48 days vs. 2.82 days vs. 2.92 days vs. 3.16 days, *P* < 0.001), hospital length of stay (6.77 days vs. 7.73 days vs. 8.21 days vs. 9.07 days, *P* < 0.001), hospital mortality (7.14% vs. 11.91% vs. 20.41% vs. 28.20%, *P* < 0.001), 28-day mortality (8.93% vs. 15.31% vs. 24.25% vs. 34.49%, *P* < 0.001) and 1-year mortality (18.52% vs. 31.29% vs. 45.13% vs. 57.42%, *P* < 0.001).

### Primary outcomes

**Figure 2:**
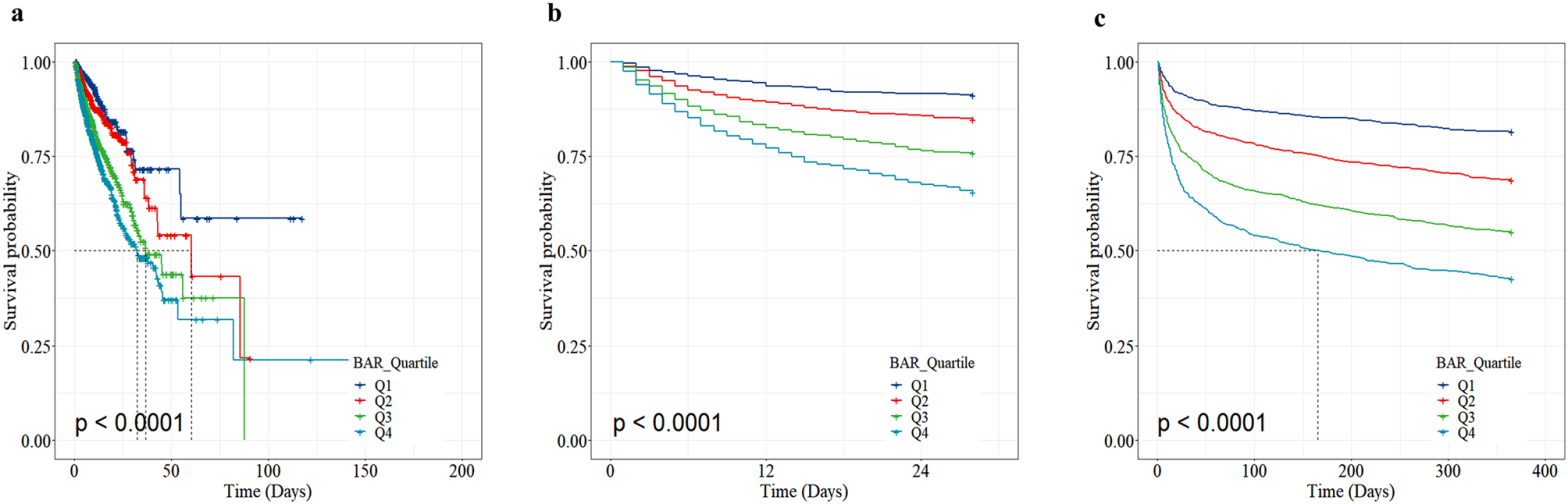
Kaplan–Meier curves illustrates the correlation between the BAR and all-cause mortality of patients with CHD. BAR, blood urea nitrogen-to-albumin ratio; CHD, coronary heart disease; BAR index quartiles: Q1, 0.55–4.98; Q2, 4.98–8.00; Q3, 8.00–14.00; Q4, 14.00–52.90. (**a**) Meier survival analysis curve for all-cause deaths in-hospital; (**b**) Meier survival analysis curve for all-cause deaths within 28-day after ICU admission; (**c**) Meier survival analysis curve for all-cause deaths within 1-year after ICU admission.

The Kaplan–Meier survival curves were analyzed to show the incidence of primary outcomes among four risk groups based on quartiles of the BAR index (Fig. 2). The log-rank test showed a statistically significant difference in in-hospital, 28-day and 1-year mortality rate among groups, respectively (*P* < 0.001). In the unadjusted Cox regression analyses, higher BAR index was found to be associated with an higher risk of in-hospital mortality, 28-day mortality and 1-year mortality. After adjusting for partly sociodemographic factors, these associations remained significant. Adjusted for full covariates, the hazard ratios (95% confidence intervals) for in-hospital mortality in higher BAR index quartiles compared to the lowest quartiles were 1.13 (0.84–1.52), 1.45 (1.09–1.94), and 1.61 (1.16–2.24), respectively. And there was a significant tendency towards an increasing CHD mortality risk across the quartiles of the BAR index (*P* for trend = 0.005). When the BAR index was a continuous variable, each 1-SD increment in the values was associated with 11% increase in the mortality risk of CHD.

In the fully adjusted model, hazard ratios (95% confidence intervals) of CHD mortality in higher quartiles versus the lowest quartiles were 1.25 (0.96–1.62), 1.60 (1.23–2.09), and 2.03 (1.51–2.74), respectively, for the 28-day follow-up, and 1.34 (1.12–1.61), 1.74 (1.44–2.10), and 2.24 (1.80–2.79), respectively, for the 1-year follow-up. The trends across quartiles were significant for both different follow-up time (both *P* for trend < 0.001). Per 1-SD increment in values of the BAR index was associated with 17% and 23% greater risk of 28-day and 1-year CHD mortality, respectively (Table 2).

**Table 2:** Cox proportional hazard ratios (HR) for all-cause mortality.

BAR index	Model 1		Model 2		Model 3	
	HR (95% CI)	*P* for trend	HR (95% CI)	*P* for trend	HR (95% CI)	*P* for trend
Hospital mortality						
Continuous variable, Per 1 SD	1.32 (1.25–1.40)		1.31 (1.24–1.39)		1.11 (1.02–1.21)	
Quartile*		< 0.001		< 0.001		0.005
Q1	Ref.		Ref.		Ref.	
Q2	1.50 (1.13–1.99)		1.41 (1.06–1.88)		1.13 (0.84–1.52)	
Q3	2.42 (1.86–3.14)		2.19 (1.68–2.85)		1.45 (1.09–1.94)	
Q4	3.05 (2.37–3.92)		2.88 (2.23–3.71)		1.61 (1.16–2.24)	
28-days mortality						
Continuous variable, Per 1 SD	1.50 (1.42–1.57)		1.47 (1.39–1.55)		1.17 (1.08–1.27)	
Quartile*		< 0.001		< 0.001		< 0.001
Q1	Ref.		Ref.		Ref.	
Q2	1.78 (1.38–2.30)		1.64 (1.27–2.11)		1.25 (0.96–1.62)	
Q3	2.98 (2.36–3.77)		2.61 (2.06–3.31)		1.60 (1.23–2.09)	
Q4	4.47 (3.57–5.61)		4.13 (3.28–5.19)		2.03 (1.51–2.74)	
1-year mortality						
Continuous variable, Per 1 SD	1.49 (1.43–1.55)		1.47 (1.41–1.53)		1.23 (1.16–1.31)	
Quartile*		< 0.001		< 0.001		< 0.001
Q1	Ref.		Ref.		Ref.	
Q2	1.83 (1.53–2.18)		1.63 (1.37–1.95)		1.34 (1.12–1.61)	
Q3	2.94 (2.49–3.47)		2.50 (2.11–2.96)		1.74 (1.44–2.10)	
Q4	4.20 (3.58–4.93)		3.80(3.23–4.47)		2.24 (1.80–2.79)	

Model 1: unadjusted;

Model 2: adjusted for age, sex, race;

Model 3: adjusted for age, sex, race, hemoglobin, platelet, glucose, WBC, bicarbonate, creatinine, ALT, heart rate, SBP, MBP, heart failure, obesity, hypertension, diabetes, AKI, sepsis, SOFA and OASIS.

*CI* confidence interval.

*BAR index: Q1, 0.55–4.98; Q2, 4.98–8.00; Q3, 8.00–14.00; Q4, 14.00–52.90.

**Figure 3:**
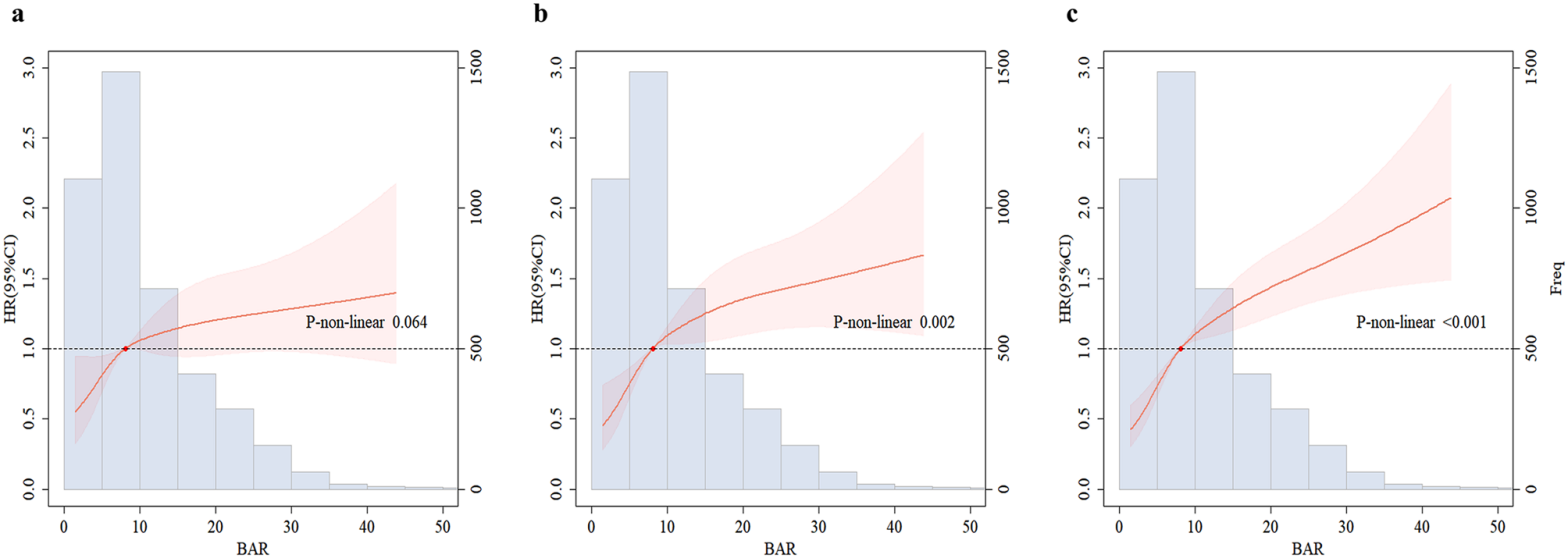
Multivariable-adjusted HR and 95% CI for primary endpoint events based on restricted cubic spines for the BAR index. (**a**) Restricted cubic spline for hospital mortality. (**b**) Restricted cubic spline for 28-day mortality. (**c**) Restricted cubic spline for 1-year mortality. *HR* hazard ratio, *CI* confidence interval, *BAR* blood urea nitrogen-to-albumin ratio.

Furthermore, multivariable-adjusted restricted cubic spline regression analyses indicated a nonlinear in the higher risk of 28-day and 1-year CHD mortality with increasing BAR index (*P* for non-linearity = 0.002 and *P* for non-linearity < 0.001, respectively), (Fig. 3). However, there was no evidence of a nonlinear association of BAR index with in-hospital CHD mortality (*P* for non-linearity = 0.064).

### Subgroup analysis

**Figure 4:**
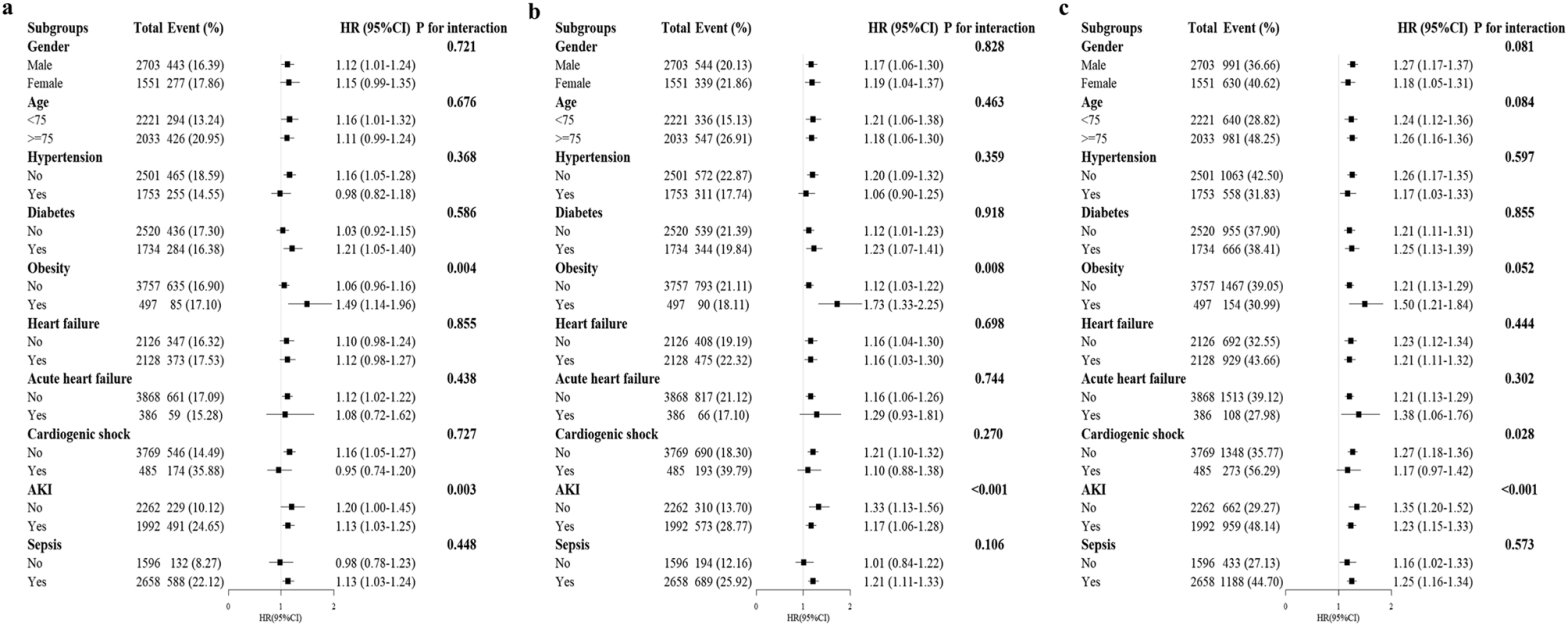
Forest plots of adjusted hazard ratios with 95% CI for the primary endpoint events in different subgroups. (**a**) Subgroup analysis for in-hospital mortality. (**b**) Subgroup analysis for 28-day mortality. (**c**) Subgroup analysis for 1-year mortality. *HR* hazard ratio, *CI* confidence interval, *AKI* acute kidney injury.

We conducted stratified analyses to investigate the relationship between the BAR index and the in-hospital, 28-day and 1-year CHD mortality in different subgroups of the enrolled patients, which included sex, age, hypertension, diabetes, obesity, heart failure, acute heart failure, cardiogenic shock (CS), acute kidney injury (AKI), and sepsis (Fig. 4). We observed that the associations between admission BAR index and mortality risk differed by the status of obesity and AKI, and the relationship between BAR and 1-year CHD mortality differed by the status of CS (*P* for interaction < 0.05). Interestingly, the BAR index appeared to have more pronounced predictive value in patients with obesity and those without AKI and CS.

### ROC curve analysis

**Figure 5:**
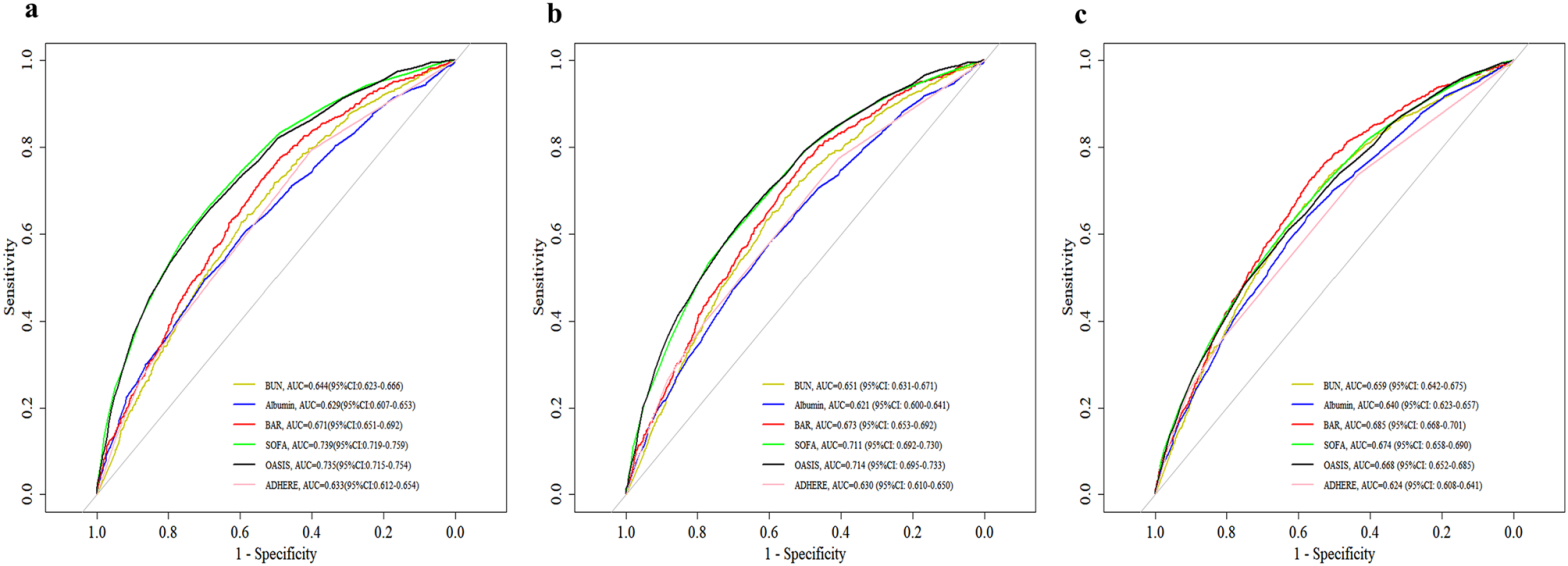
ROC curves for the prediction of in-hospital, 28-day, and 1-year all-cause mortality of BUN, albumin, BAR, SOFA score, OASIS score and ADHERE score. (**a**) ROC curves for in-hospital mortality. (**b**) ROC curves for 28-day mortality. (**c**) ROC curves for 1-year mortality. *ROC* receiver operating characteristic, *BUN* blood urea nitrogen, *BAR* blood urea nitrogen-to-albumin ratio, *SOFA* sepsis-related organ failure assessment, *OASIS* oxford acute severity of illness, *ADHERE* acute decompensated heart failure national registry.

ROC curves for BUN, albumin, BAR, SOFA, OASIS, and ADHERE scores in predicting in‐hospital mortality, as well as mortality within 28-day and 1-year post-ICU admission are depicted in Fig. 5. The AUCs of the BAR index in predicting in-hospital, 28-day, and 1-year mortality were 0.671, 0.673, and 0.685, respectively. Notably, the AUCs of the BAR index were better than those for BUN and albumin, or the ADHERE score at all scenarios (*P* < 0.05). Although the predictive performance of the BAR index for in-hospital and 28-day mortality was lower than that of the SOFA and OASIS scores (*P* < 0.05), our findings indicated that the BAR index exhibited an incremental predictive advantage for longer-term mortality outcomes. In particular, for predicting 1-year mortality, the BAR index was found to have a higher predictive ability than both SOFA and OASIS scores, albeit without statistical significance (*P* > 0.05). We also determined the optimal cut-off value of 7.36 for the BAR index in predicting 1-year mortality, achieving a sensitivity of 72.50% and a specificity of 56.70%.

## Discussion

Even after adjusting for sociodemographic factors and other clinical covariates, our study found the higher BAR index continued to exhibit a significant association with higher incidence of in-hospital mortality, 28-day mortality and 1-year mortality in coronary heart disease patients in the ICU.

As the ratio involving both BUN and serum albumin, BAR could be assessed as a comprehensive body reserves. It encompasses malnutrition, dehydration, liver and kidney functionality, which may enhance the utility in assessing disease severity^18^. Some studies have mainly explored the relationships between BAR and the prognosis of patients in sepsis and respiratory diseases, with limited investigations into its association with CVD. Lin et al. proposed the utility of BAR as a convenient predictor for short-term prognostication among critically ill patients with chronic heart failure, potentially offering an alternative to the SOFA score^19^. Similarly, another study that enrolled 1827 patients indicated that BAR > 7.83 was associated with 4-year mortality in patients with AMI^20^. Wang et al. found that a significant positive association between BAR and all-cause mortality among sepsis patients, exhibiting the best predictive value with a higher area under the curve than BUN and albumin individually^17^. Ugajin et al. conducted a prospective study and they identified that an increased BAR serves as a simple yet independent predictor of both mortality and the severity of community-acquired pneumonia^21^. Our study served as a valuable complement to the existing literature by focusing specifically on patients with CHD, demonstrating that the BAR index possessed higher predictive capability compared to BUN and albumin alone. Furthermore, we found that the BAR index had a greater predictive advantage for longer-term outcomes comparable to the SOFA and OASIS scores in predicting 1-year mortality. These findings suggested that the BAR index may serve as a valuable tool for risk stratification.

In this specific cohort of ICU patients with CHD, our study demonstrated a progressive increase in both ICU and hospital length of stay, as well as higher hospital mortality, as the BAR index values increase. These outcomes undoubtedly place substantial burdens on both families and society as a whole. Subgroup analyses shed light on the differential impact of the BAR index in specific patient populations. Notably, the predictive value of the BAR index was more pronounced in patients with obesity and those without AKI or CS. This variation in outcomes might be explained by reverse causality, patients previously diagnosed with AKI or CS might have received effective treatment or adopted healthier lifestyles, potentially mitigating the risk of adverse outcomes^22^. Consequently, clinicians may utilize the BAR index as a valuable tool for the early identification of high-risk patients, facilitating a more targeted and proactive approach to their management.

The exact causes and underlying pathophysiological mechanisms of the association between the BAR index and adverse outcomes remain unclear^23^. Therefore, we can only speculate on possible mechanisms by focusing on the primary components of the BAR index, namely bun and albumin. The relationships between bun and CVD, as well as albumin and CVD, had been studied. Blood urea nitrogen plays a pivotal role in assessing renal function. The elevation of BUN concentration is not only solely influenced by the fall in glomerular filtration rate, but also depends on the degree of urea reabsorption^24,25^. Urea reabsorption occurs passively in conjunction with the reabsorption of sodium and water which is regulated by antidiuretic hormone^25^ under the influence of angiotensin-II^26^. Thus, an elevated BUN likely indicate a suitable kidney reaction to renal hypoperfusion in the context of the hypovolemia, renovascular disease, or reduced cardiac output^5,6^. BUN is also proposed as the indicator of neurohormonal activation^27^. Elevation in BUN reflects the combined impact of hemodynamic and neurohormonal alterations^28^. The reabsorption process of BUN in the tubules may be influenced by the activation of the renin–angiotensin–aldosterone system, sympathetic nervous activity and arginine-vasopressin activity^26,29^, which are recognized factors associated with cardiovascular risk. Some previous studies have reported that low albumin is correlated with poor prognosis in a variety of cardiovascular disease^2,19,30^. The potential impact of decreased serum albumin levels in CVD may be attributed to its abilities as an anti-inflammatory, antioxidant, and antithrombotic activities^31–35^. Reduced levels of serum albumin might contribute to higher blood viscosity, impairment of endothelial functions, and a decline in antioxidant capacity. These combined effects could potentially heighten the vulnerability to atherothrombosis, consequently resulting in adverse outcomes^36,37^. In addition, hypoalbuminemia can exacerbate conditions like pulmonary edema and fluid retention, further contributing to the deterioration of ischemic heart disease^32^.

A main strength of our study lies in the finding that a higher BAR index serves as a independent predictor of greater mortality among patients with CHD in the ICU. Our study drew on data from the extensive and diverse population within the MIMIC-IV database. The wealth of patient data in this database allowed us to conduct strict statistical adjustments, mitigating the influence of potential confounding factors.

Nevertheless, our study is subject to some limitations. First, we only considered the values of BUN and serum albumin at ICU admission without assessing the dynamic variations during their hospital stay. Second, some variables were excluded from our analysis due to missing data, like triglycerides and c-reactive protein, which may be helpful to elucidate the underlying mechanism of CHD mortality. Third, owing to the lack of certain necessary variables for computing the GRACE (Global Registry of Acute Coronary Events) score, we were unable to calculate it and unable to compare the BAR index to the GRACE score in our study^38^. Finally, this prospective study could not establish causality conclusively. Despite multivariate adjustment and subgroup analyses, there are still some other potential confounding factors might affect the results.

## Conclusion

In summary, our study demonstrated that BAR index was an objective predictor for both short-term and long-term mortality among patients with CHD in the ICU. Utilizing the BAR index as a measurement could aid in categorizing risk and predicting prognosis for patients with CHD. Furthermore, our findings can serve as a foundation for the future studies in diverse clinical settings.

## Methods

### Database and study population

The data in this study were all obtained from a large publicly accessible MIMIC-IV database version 2.2 (https://mimic.physionet.org/about/mimic/), which is the latest version that encompasses extensive clinical data from patients admitted to the Beth Israel Deaconess Medical Center between 2008 and 2019. MIMIC-IV contains more than 50,000 admissions information for adult patients including demographic characteristics, laboratory tests results, medication treatment, vital signs and other comprehensive information^39^. To obtain access, one author (ZLZ) completed web-based training courses and exams and gained full access to the database (certification number: 53158939). The collection of patient information and creation of the MIMIC-IV data was reviewed by the Institutional Review Board at the Beth Israel Deaconess Medical Center, who granted a waiver of informed consent and approved the data sharing initiative.

This study enrolled patients who had been diagnosed with CHD, which was defined as the occurrence of myocardial infarction (MI), acute coronary syndrome, ischemic heart disease, or those who had undergone percutaneous coronary intervention or coronary artery bypass grafting^40^. These diagnosis were based on the International Classification of Diseases (ICD) codes, specifically ICD-9 code “410”, “411”, “413”, “414” and ICD-10 code “I20”, “I21”, “I22”, “I24”, “I25”. The exclusion criteria were as follows: (1) patients who were admitted to the ICU multiple times for CHD, only the data of their initial admission were retained for analysis (n = 16,145); (2) patients aged < 18 years at the time of the first admission; (3) patients with an ICU length for less than 24 h; (4) patients without recorded (blood urea nitrogen and albumin) within 24 h of admission. Finally, 4254 patients were enrolled in our study and categorized into four groups according to the quartiles of the BAR index.

### Data extraction

The PostgreSQL software (version 15.2) were used to extract data by running Structured Query Language (SQL) from the MIMIC-IV database. The extraction of potential confounders could be categorized into following primary groups, including (1) Demographics: age, gender, race, weight and height; (2) Vital Signs: heart rate, systolic blood pressure, diastolic blood pressure, mean arterial pressure and respiratory rate; (3) Comorbidities: myocardial infarct, heart failure, acute kidney injury (AKI), sepsis, respiratory failure, hypertension, diabetes and obesity; (4) Disease Severity Scores: the sepsis-related organ failure assessment score (SOFA), simplified acute physiology score II (SAPSII), systemic inflammatory response syndrome (SIRS), Oxford acute severity of illness score (OASIS) and Acute Decompensated Heart Failure National Registry (ADHERE score, calculate by utilizing blood urea nitrogen, systolic blood pressure and creatinine)^41^; (5) Laboratory Indicators: hemoglobin, platelets, white blood cells (WBC), anion gap, bicarbonate, serum sodium, serum calcium, serum potassium, serum chloride, total bilirubin, serum glucose, serum creatinine, international normalized ratio (INR), prothrombin time (PT), partial thromboplastin time (PTT), alanine aminotransferase (ALT), alkaline phosphatase (ALP) and aspartate aminotransferase (AST). The value of the BAR index was the ratio of bun and albumin. Furthermore, the primary outcomes of this study included in-hospital mortality, all-cause mortality within 28-day and 1-year after ICU admission. These outcomes were calculated based on the hospital stay data, like the hospital expire flag, and the follow-up data, such as the recorded out-of-hospital date of death. All vital signs, laboratory variables and disease severity scores were obtained from the data collected within the initial 24 h after the patient's admission to the ICU.

In order to avoid potential bias, variables were excluded in the analysis if they had missing values exceeding 15% of the total observations. Variables with missing values less than 15% were processed by multiple imputation using the Predictive Mean Matching (PMM) method through the “mice” package in R^42^.

### Statistical analysis

Continuous variables were presented as median (Interquartile range), whereas categorical variables were expressed as frequencies and proportions. The former were compared by Wilcoxon test or Kruskal–Wallis test, and the latter were compared by Pearson’s chi-squared test. Kaplan–Meier survival analysis was used to assess the relationship between the incident of CHD during follow-up and the quartiles of the BAR index, differences among groups were assessed by log-rank tests.

Cox proportional hazard models were used to estimate hazard ratios (HR) and 95% confidence intervals (CI) for the higher quartiles compared to the lowest quartile, as well as for each 1-standard deviation increment in the BAR index for all the study endpoints. The *P* values for liner trend were calculated based on the median value of each category of BAR index as a continuous variable. Three models were constructed to sequentially adjust for potential confounders of CHD: model 1: unadjusted; model 2: adjusted for age, sex, and race; model 3: adjusted for age, sex, race, hemoglobin, platelet, glucose, WBC, bicarbonate, creatinine, ALT, heart rate, SBP, MBP, heart failure, obesity, hypertension, diabetes, AKI, sepsis, SOFA and OASIS. The proportional hazards assumption was assessed relying on Schoenfeld residuals, the results revealed that there were no violations with a *P* value > 0.05. Additionally, we investigated the possible nonlinear relationship between the baseline BAR index and the risk of all-cause mortality using a restricted cubic spline (RCS) regression model with four knots. Subgroup analyses were conducted to explored whether the associations varied based on sex, age (< 75, ≥ 75 years), hypertension, diabetes, obesity, heart failure, acute heart failure, CS, AKI and sepsis. Tests for the interactions between the factor of interest and each potential effect modifier were performed by fitting an interaction term.

We conducted Receiver Operating Characteristic (ROC) analysis to assess the predictive power of BUN, albumin, BAR, SOFA, OASIS, and ADHERE regarding in-hospital mortality, as well as mortality within 28 days and 1 year following ICU admission. To evaluate the discriminative performance of these parameters, we employed the DeLong test to compare their areas under the curves (AUCs). Additionally, the optimal cut-off value for BAR was identified using the Youden index, optimizing the balance between sensitivity and specificity.

All statistical analyses were performed by using R software (version 4.2.3). A two-sided *P* value of < 0.05 was considered statistically significant.

## Data Availability

The data that support the findings of the study are available through the website of MIMIC-IV: https://mimic.physionet.org/about/mimic/.
